# TissueGene-C promotes an anti-inflammatory micro-environment in a rat monoiodoacetate model of osteoarthritis via polarization of M2 macrophages leading to pain relief and structural improvement

**DOI:** 10.1007/s10787-020-00738-y

**Published:** 2020-07-21

**Authors:** Hyeonyoul Lee, Heungdeok Kim, Jinwon Seo, Kyoungbaek Choi, Yunsin Lee, Kiwon Park, Sujeong Kim, Ali Mobasheri, Heonsik Choi

**Affiliations:** 1 Institute of Bio Innovation Research, Kolon Life Science, Inc., Magok-dong, Gangseo-gu, Seoul, Korea; 2 Department of Regenerative Medicine, State Research Institute Center for Innovative Medicine, Santariskiu 5, 08406 Vilnius, Lithuania; 3 Research Unit of Medical Imaging, Physics and Technology, Faculty of Medicine, University of Oulu, 90014 Oulu, Finland; 4 Department of Orthopedics and Department of Rheumatology and Clinical Immunology, University Medical Center Utrecht, 508 GA Utrecht, The Netherlands; 5 Centre for Sport, Exercise and Osteoarthritis Research Versus Arthritis, Queen’s Medical Centre, Nottingham, NG7 2UH UK

**Keywords:** TissueGene-C, Osteoarthritis, Anti-inflammatory, Cell therapy, Gene therapy, M2 macrophage

## Abstract

Osteoarthritis (OA) is the most common form of arthritis, characterized by cartilage destruction, pain and inflammation in the joints. Existing medications can provide relief from the symptoms, but their effects on the progression of the disease are limited. TissueGene-C (TG-C) is a novel cell and gene therapy for the treatment of OA, comprising a mixture of human allogeneic chondrocytes and irradiated cells engineered to overexpress transforming growth factor-β1 (TGF-β1). This study aims to investigate the efficacy and mechanism of action of TG-C in a rat model of OA. Using the monosodium-iodoacetate (MIA) model of OA, we examined whether TG-C could improve OA symptoms and cartilage structure in rats. Our results showed that TG-C provided pain relief and cartilage structural improvement in the MIA OA model over 56 days. In parallel with these long-term effects, cytokine profiles obtained on day 4 revealed increased expression of interleukin-10 (IL-10), an anti-inflammatory cytokine, in the synovial lavage fluid. Moreover, the increased levels of TGF-β1 and IL-10 caused by TG-C induced the expression of arginase 1, a marker of M2 macrophages, and decreased the expression of CD86, a marker of M1 macrophages. These results suggest that TG-C exerts a beneficial effect on OA by inducing a M2 macrophage-dominant micro-environment. Cell therapy using TG-C may be a promising strategy for targeting the underlying pathogenic mechanisms of OA, reducing pain, improving function, and creating a pro-anabolic micro-environment. This environment supports cartilage structure regeneration and is worthy of further evaluation in future clinical trials.

## Introduction

Osteoarthritis (OA) is a progressive, degenerative, low-grade inflammatory disease characterized by cartilage extracellular matrix (ECM) degradation, synovitis, and structural modification of the subchondral bone, most commonly in the knee and hip joints. The main symptom of OA is pain, which leads to functional limitations that severely impact the quality of life of patients (Felson 2006; Hunter and Bierma-Zeinstra 2019; Loeser et al. 2012). Currently, OA affects more than 250 million people globally. Aging and obesity are known risk factors for OA, so its prevalence is expected to rapidly increase as the average life expectancy of the population increases and lifestyles become increasingly sedentary, thereby promoting obesity (Lee et al. 2013; Sharma et al. 2006; Wallace et al. 2017). Despite the high prevalence of OA, there are no effective disease-modifying osteoarthritis drugs (DMOADs) that can relieve pain, repair damaged cartilage, and support the healing of the surrounding joint tissues (Oldershaw 2012; Perera et al. 2012). Analgesia is the traditional treatment for OA, but it is insufficient in treating the structural modifications or altering the progress of the disease as analgesic drugs cannot reduce inflammation or halt/reverse cartilage damage (Appleton 2018). To overcome these limitations and develop more effective therapeutics, recent strategies have focused on the employment of chondrogenic growth or anti-inflammatory factors (Li et al. 2017).

OA is conventionally considered a “wear and tear” disease. However, recent research has demonstrated the importance of low-grade inflammation and chronic synovial inflammation in the development and symptomatic progression of OA. It has been shown that mechanically driven inflammation precedes detectable cartilage degeneration in OA (Roemer et al. 2011; Sokolove and Lepus 2013) and that activated synovial macrophages appear to play a role in OA pathogenesis (Bondeson et al. 2006; Goldring 1999; Pelletier et al. 1991, 2001). Thus, there are a number of ongoing efforts to treat OA with anti-inflammatory strategies designed to block pro-inflammatory cytokines or modify macrophage phenotype and function (Bondeson 2010; Bondeson et al. 2010; Mosser and Edwards 2008). Activated macrophages may exhibit 2 phenotypes: M1 macrophages that have pro-inflammatory properties and M2 macrophages that exhibit immunosuppressive properties (Mosser and Edwards 2008). The induction of M2 macrophages improves the micro-environment supporting tissue repair and regeneration in inflammatory diseases, such as OA. Transforming growth factor-β1 (TGF-β1) has been shown to possess potent immunosuppressive and anti-inflammatory properties through the regulation of the ability of monocytes/macrophages to release inflammatory cytokines (Becker et al. 2006; Zhang et al. 2016). Additionally, it positively regulates chondrocyte proliferation, differentiation, and ECM synthesis and deposition (Blaney Davidson et al. 2007; van Beuningen et al. 1994; Yang et al. 2001). Currently, there are no therapeutic strategies that incorporate cell and gene therapy with TGF-β1 as the key biological component.

TissueGene-C (TG-C) is a novel gene and cell therapy consisting of human allogeneic chondrocytes and irradiated GP2-293 cells overexpressing TGF-β1 mixed in a 3:1 ratio (Noh et al. 2010; Yoon et al. 2015). Clinical trials have demonstrated that TG-C treatment improves pain and function in patients with knee OA (Cherian et al. 2015; Ha et al. 2015). Moreover, TG-C reduces inflammation while boosting the intrinsic anabolic function of chondrocytes. These observations support TG-C as a DMOAD candidate. However, despite the remarkable results of these clinical trials, the molecular mechanisms underlying the effect of TG-C remain elusive. The aim of this study was to verify the potential of TG-C as a DMOAD and elucidate its mechanism of action, particularly its impact on macrophage polarization, in a well-established preclinical model of OA. In this study, we investigated whether TG-C can induce pain relief and improve the cartilage structure in arthritic knee joints by stimulating the polarization of M2 macrophages using a rat monoiodoacetate (MIA) OA model.

## Materials and methods

### Animals

**Fig. 1:**
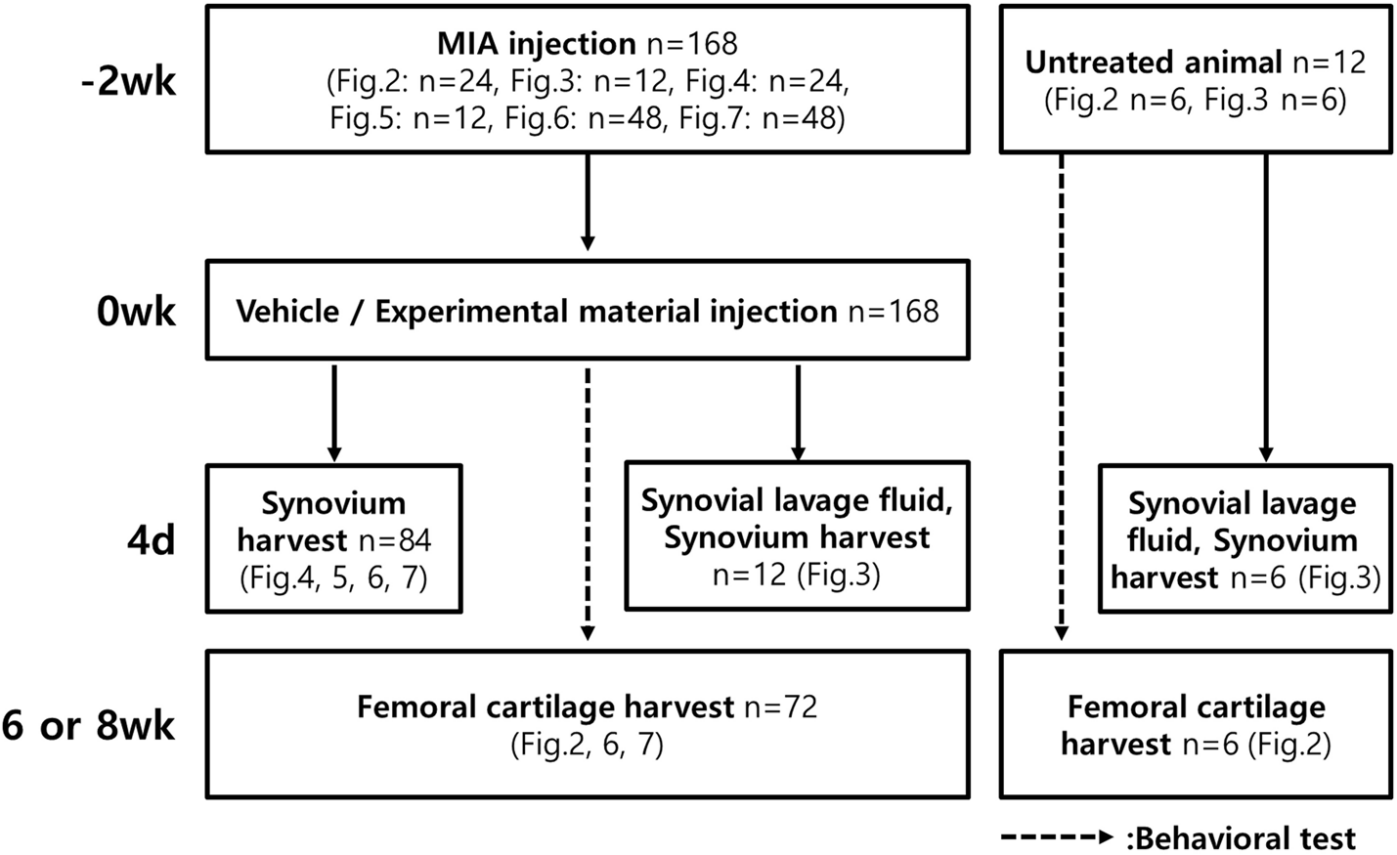
Experimental design. Behavioral test, histological evaluation, immunohistochemical evaluation, and molecular analysis were performed. Each experiment was not performed simultaneously, but proceeded sequentially.

A total of 180 6-week-old male Sprague–Dawley rats (200–225 g) were obtained from Nara Biotech, Inc (Pyeongtaek, Korea). The number of animals used for each experiment is shown in Fig. 1. All animal experiments were conducted under appropriate veterinary supervision at the animal facility of Kolon Life Science, Inc. (Seoul, Korea) with approval from the Institutional Animal Care and Use Committee (IACUC No. KLS IACUC-2014-02, 2014-08, 2016-09, 2016-10, 2016-17, 2016-22). Animals were maintained in a temperature- and humidity-controlled colony on a 12-h light/dark cycle and acclimatized to this environment for 1 week before experimentation. All studies involving animals were carried out in accordance with the ARRIVE guidelines (Kilkenny et al. 2010; McGrath et al. 2010).

### Induction of OA and injection of cells

MIA (3 mg; Sigma Aldrich, Mo, USA) was diluted in 50 μL of saline and intra-articularly injected into the left knee of 7-week-old rats using a 31 G syringe. Two-week after MIA injection, when the OA micro-environment was sufficiently induced, 50 μL of human allogeneic chondrocytes (HC, 9.0 × 10^5^ cells), irradiated GP2-293 cells expressing TGF-β1 (TC, 3.0 × 10^5^ cells), TG-C (1.2 × 10^6^ cells, mixture of HC [9.0 × 10^5^ cells] and TC [3.0 × 10^5^ cells]) or the vehicle (Cryostor^®^ CS-10, BioLifeSolution, WA, USA) were administered to the left knee joint cavity of rats, except those in the untreated normal group. General symptoms were monitored and individual body weight was measured once a week for each group (*n* = 6/group).

### The *von Frey* filament test

Dixon’s 50% up-down threshold method (Dixon 1980) was used in this study. Briefly, each rat was placed on a metal mesh and allowed to acclimatize for 10 min. The *von Frey* filaments (Stoelting Co, IL, USA) were applied in ascending order of force using a series of monofilaments that ranged from 0.4 to 15 g to the mid-plantar area of the left hind paw. A positive response was defined as a rapid withdrawal or licking of the paw. Testing began with the 2 g monofilament. If the rat responded to the first filament, the next lower filament was used until the rat stopped exhibiting a positive response or a response to the lowest filament (0.4 g) was observed. If a rat did not show a positive response, the next higher filament in the sequence was tested until the rat showed a positive response or no response to the highest filament (15 g) was observed. The threshold was determined according to the standard provided by Dixon (1980). Each test was conducted by three independent, blinded observers.

### Histological analysis and microscopic scoring of OA lesions

At day 42 or 56 post-treatment, rats were anesthetized with 5% isoflurane before being killed using carbon dioxide. Knee joints were dissected and fixed in 10% neutral buffered formalin (BBC BioChemical, WA, USA). After fixation, joints were decalcified in RapidCal-Immuno™ (BBC BioChemical) and processed for histology. Tissue samples were embedded in paraffin and sectioned into 3 µm thick pieces for hematoxylin and eosin (H&E) (BBC BioChemical) and Masson’s trichrome (Sigma Chemical Co) staining. To evaluate the rat OA model, histological assessment was conducted, using the modified Mankin’s scores system for cartilage degeneration, and graded 0, 1, 2, and 3, which correspond to mild, moderate, and severe. Each experimental group was scored by the summation of individual grades. The severity of OA lesions was graded by three independent blind observers (Pearson et al. 2011).

### Immunohistochemistry

At day 4 post-treatment, the synovial membranes embedded in paraffin blocks were cut into 3-µm-thick sections and mounted on glass microscope slides. Immunohistochemical (IHC) staining was performed using anti-CD86 antibody (Abcam, MA, USA) or anti-arginase 1 antibody (BD Biosciences, CA, USA). Femoral epiphyseal cartilage was embedded in paraffin, sectioned to 3 µm and mounted on glass microscope slides. Immunohistochemical staining was performed using anti-collagen type I antibody (Merck Millipore, MA, USA) or anti-collagen type II antibody (Merck Millipore, MA, USA). Slides were processed with 3% H_2_O_2_, blocked with serum for 1 h at 25 °C, incubated with the primary antibody at 4 °C overnight, and processed using a standard avidin–biotin immunohistochemical assay as per the manufacturer’s instructions (Invitrogen, CA, USA). Diaminobenzidine (DAB) was used as a chromogen (Vector, CA, USA), and commercial hematoxylin (Invitrogen) was used for counterstaining (Lee et al. 2001). The slides were examined microscopically (Axio Scope. A1, Carl Zeiss, Germany), and the immunostained cells were counted with Image-Pro^®^ Plus 7.0 (Media Cybernetics Inc., MD, USA).

Double immunofluorescence (IF) staining was performed for the synovial membrane tissues with anti-CD68 (AbD serotec, NC, USA) and anti-IL-10 (Biorbyt, UK) antibodies. Tissue sections were incubated at 4 °C overnight with the primary antibodies, followed by incubation with the secondary antibodies. The anti-CD68 antibodies were incubated with Alexa Fluor^®^ 594 chicken anti-rabbit IgG (Life Technologies, CA, USA), and the anti-IL-10 antibodies were incubated with Alexa Fluor^®^ 488 goat anti-IgG (Life Technologies) for 1 h at room temperature, followed by staining with 4′,6′-diamidino-2-phenylindole hydrochloride (DAPI) (Vector).

### Luminex platform measurement of cytokine levels

At day 4 post-treatment, 50 μL of sterile saline was injected into the joint cavity and then collected for cytokine profiling (Lu et al. 2013). Concentrations of IL-10 in the synovial lavage fluid were measured using a rat cytokine magnetic bead panel as per the manufacturer’s instructions (Merck Millipore). Data analysis was performed with xPONENT 3.1 (Luminex, TX, USA).

### Preparation of RNA and quantitative RT-PCR

Total RNA was isolated at day 4 post-treatment from rat synovial membranes using the RNeasy Lipid Tissue Mini kit (QIAGEN, CA, USA) and cDNA was synthesized using the SuperScript™ III First-Strand Synthesis System (Invitrogen) as per the manufacturer’s instructions. The polymerase chain reaction (PCR) mixture was prepared to a final volume of 50 µL, as follows: 1 µL cDNA, 0.2 µM of each primer, 10 µL SYBR Premix Ex Taq (TAKARA Bio, Shiga, Japan). For a total of 40 cycles, the reaction occurred under the following conditions: 10 s at 95 °C and 30 s at 60 °C. Expression levels of the target genes were quantified with the ABI 7900 real-time PCR system (RT-PCR, Applied Biosystems, CA, USA). The relative expression levels were analyzed using the $2^{{ - \Delta \Delta C_{{\text{T}}} }}$ method by normalizing against the housekeeping gene β-actin. Primer sequences used for quantitative RT-PCR (qPCR) are shown in Table 1.

**Table 1:** Primer sequences for qRT-PCR analysis of gene expression in rats

Gene name	Forward primer	Reverse primer
CD86	TCCTCCAGCAGTGGGAAACA	TTTGTAGGTTTCGGGTATCCTTGC
CD163	CTCAGCGTCTCTGCTGTCAC	GGCCAGTCTCAGTTCCTTCTT
Arg1	TTGATGTTGATGGACTGGAC	TCTCTGGCTTATGATTACCTTC
IL-1β	TCCAGGATGAGGACCCAAGC	TCGTCATCATCCCACGAGTCA
TNFα	ACTGAACTTCGGGGTGATTG	GCTTGGTGGTTTGCTACGAC
IL-10	CAAGGCAGTGGAGCAGGTGA	CCGGGTGGTTCAATTTTTCATT
Hmox-1	AGAGTTTCCGCCTCCAACCA	CGGGACTGGGCTAGTTCAGG
IL10Ra	CTGGTCACCCTGCCATTGAT	AGGCATGGCCAAAATACAAAGAAAC
CD68	ACTGGGGCTCTTGGAAACTACAC	CCTTGGTTTTGTTCGGGTTCA
β-actin	AGTTCGCCATGGATGACGAT	AAGCCGGCCTTGCACAT

*CD* cluster of differentiation, *Arg* arginase, *IL* interleukin, *TNFα* tumor necrosis factors alpha, *Hmox* heme oxygenase

### Neutralization of IL-10 and TGF-β1

To evaluate the roles of IL-10 and TGF-β1 in the efficacy of TG-C, rats were injected with anti-IL-10 antibody (Abcam) or anti-TGF-β1 antibody (Abcam) in the knee joints to inhibit the activity of the target protein at day 0 and at day 3 post-cell treatment. All antibodies were diluted to 500 ng/30 µL in sterile PBS and injected using the same protocol described above for the cell injection. To exclude the influence of the antibody, a control group received an isotype-matched irrelevant Ig antibody (Abcam).

### Statistical analysis

Statistical analysis was performed using Sigma plot (version 13.0) software. Data are presented as mean ± SEM (standard error of the mean) of independent samples. For behavioral tests, statistical significance between groups was determined by one-way analysis of variance, and Dunnett’s test was applied as a post hoc test if statistical significance was obtained. For qPCR analysis, statistical significance between experimental groups was determined by Student’s *t* test. The difference was considered statistically significant if *P* < 0.05 (*) or < 0.001 (**).

## Results

### TG-C induces pain relief and regenerates type II collagen-positive cartilage in a rat MIA-induced OA model

**Fig. 2:**
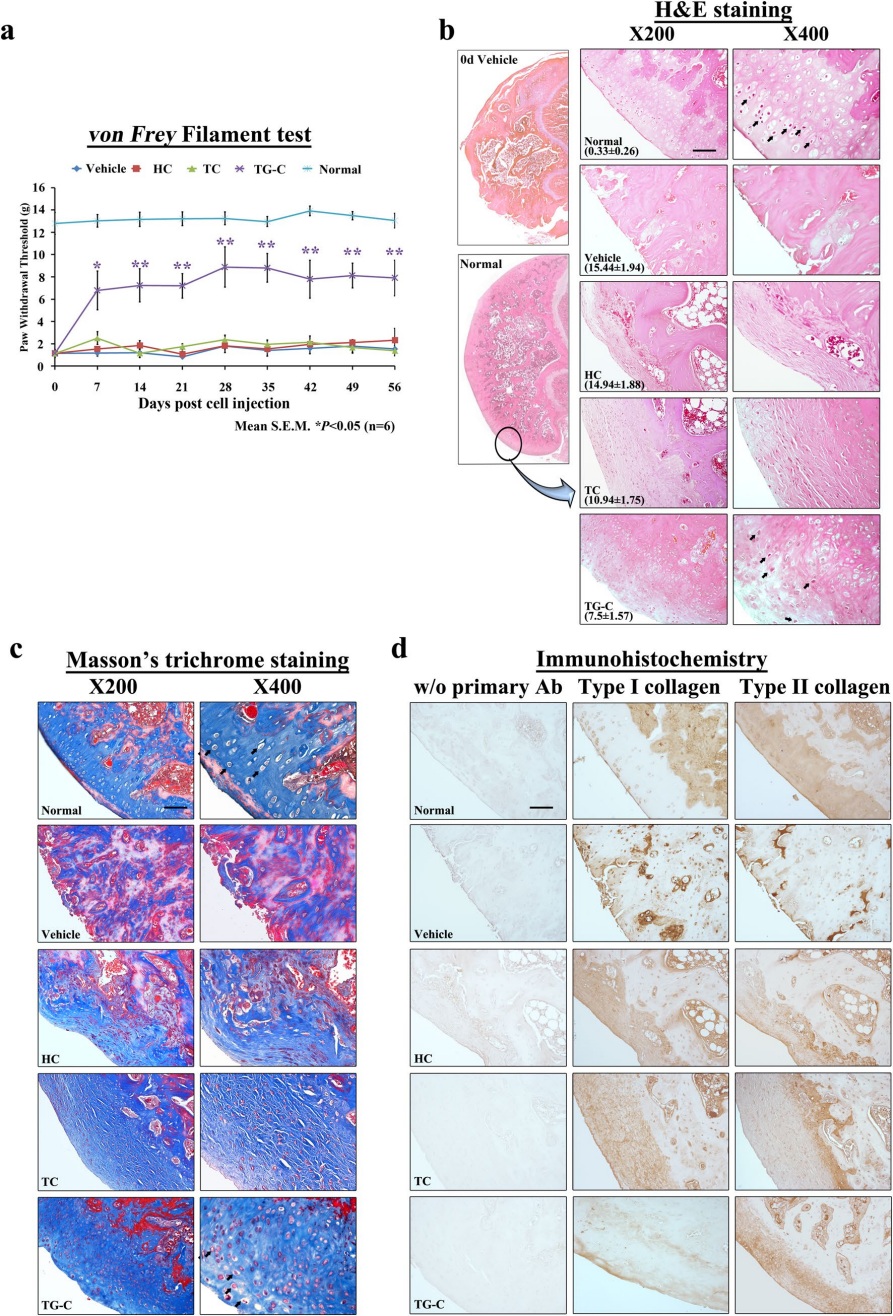
TG-C induces pain relief and promotes cartilage regeneration in the MIA-induced OA rat model. After 2 weeks post-MIA treatment, TG-C, the vehicle or each component of TG-C (HC; human allogeneic chondrocytes, TC; irradiated GP2-293 cells expressing TGF-β1) was injected into knee joints. **a** The *von Frey* filament test showed that the TG-C group exhibited pain relief. Representative results from three independent tests are shown (*n* = 30, 6 rats per group). The difference was considered statistically significant if *P* < 0.05 (*) or *P* < 0.001 (**), from the vehicle. **b** 2 weeks post-MIA injection, the degree of femoral cartilage degeneration was evaluated by H&E staining and Mankin’s score system (normal: 0.4 ± 0.32, MIA: 18,78 ± 1.61). The cartilage regeneration effect was evaluated 56 days after treatment. **c** Masson’s trichrome staining and **d** type I and II collagen immunostaining of the joint also showed similar results in cartilage regeneration. Arrows indicate chondrocytes with lacuna, representative feature of hyaline cartilage. All the histology data are representative of three independent experiments, which were harvested at day 56 post-TG-C treatment. The scale bar indicates 100 μm

14 days after the intra-articular injection of MIA, TG-C was administrated. Since pain is the principal symptom of OA (Felson 2006), we measured the mechanical allodynia reaction, which is pain caused by a stimulus that does not normally elicit pain, using the *von Frey* filament test in a MIA-induced OA model after TG-C treatment (Fig. 2a). The TG-C-treated rats (purple, Fig. 2a) experienced symptomatic pain relief relative to the vehicle-treated rats starting as early as day 7 and this was maintained until the end of the observation period (day 56 post-treatment) (day 7; vehicle vs TG-G, 1.17 ± 0.27 vs 6.79 ± 1.74; *P* < 0.05, day 56; 1.53 ± 0.13 vs 7.90 ± 1.60; *P* < 0.001; Fig. 2a). However, the HC (red) or TC (green) group did not show any pain relief (Fig. 2a) compared with the vehicle group.

We evaluated the degree of cartilage regeneration 56 days after TG-C treatment. The vehicle group showed loss of chondrocytes and cartilage structure in the femur and exposure of the subchondral bone, which are representative structural features of OA (vehicle, Fig. 2b, c). Even on day 0 of treatment, the vehicle group exhibited a pronounced disruption of cartilage structure compared to the normal group (upper left, Fig. 2b). The TG-C and TC groups showed regeneration of cartilage tissue compared with the vehicle group. By contrast, the HC group showed no cartilage structural improvement (Fig. 2b, c). Accordingly, the IHC staining revealed that the TG-C-treated group had a high proportion of type II collagen in the cartilage tissue, while the TC group exhibited type I collagen-rich cartilage (Fig. 2d). These results demonstrate that the cartilage tissue induced by TG-C was closer to normal than those induced by other treatments (Table 2). These results suggest that TG-C induces pain relief and promotes structural improvement in the knee joint of the MIA-induced OA rat model.

**Table 2:** Joint tissue pathology scores

Treatment no. of animals	Normal 6 (× 3)	Vehicle 6 (× 3)	HC 6 (× 3)	TC 6 (× 3)	TG-C 6 (× 3)
Structural change in the joint					
Surface irregularities					
1	2/18	3/18	0/18	5/18	7/18
2	0/18	4/18	1/18	2/18	4/18
3	0/18	10/18	14/18	6/18	4/18
Average pathology score	0.11	2.28	2.44	1.50	1.50
Ulceration					
1	1/18	3/18	3/18	8/18	7/18
2	0/18	8/18	7/18	6/18	6/18
3	0/18	7/18	7/18	3/18	0/18
Average pathology score	0.05	2.22	2.11	1.61	1.06
Fibrillation of cartilage surface					
1	0/18	3/18	3/18	1/18	3/18
2	0/18	2/18	6/18	6/18	5/18
3	0/18	7/18	5/18	6/18	1/18
Average pathology score	0	1.56	1.67	1.72	0.89
Disorganization of chondrocytes					
1	1/18	1/18	7/18	9/18	6/18
2	0/18	10/18	5/18	8/18	7/18
3	0/18	5/18	4/18	0/18	0/18
Average pathology score	0.05	2.00	1.61	1.39	1.11
Exposure of subchondral bone					
1	0/18	7/18	6/18	4/18	6/18
2	0/18	1/18	0/18	4/18	1/18
3	0/18	8/18	8/18	0/18	0/18
Average pathology score	0	1.83	1.67	0.67	0.44
Cellular changes of chondrocyte					
H&E staining					
1	2/18	7/18	6/18	9/18	9/18
2	0/18	2/18	3/18	4/18	1/18
3	0/18	4/18	3/18	1/18	2/18
Average pathology score	0.11	1.28	1.17	1.11	0.94
Degeneration/necrosis					
1	0/18	3/18	2/18	5/18	6/18
2	0/18	4/18	8/18	5/18	4/18
3	0/18	11/18	8/18	5/18	0/18
Average pathology score	0	2.44	2.33	1.67	0.78
H&E staining					
Reduction of staining in cartilage					
1	0/18	5/18	4/18	10/18	10/18
2	0/18	5/18	5/18	5/18	2/18
3	0/18	6/18	7/18	1/18	0/18
Average pathology score	0	1.83	1.94	1.28	0.78
Total pathology score	0.33 ± 0.26	15.44 ± 1.94	14.94 ± 1.88	10.94 ± 1.75	7.5 ± 1.57

0: normal 1: slight, 2: moderate, 3: severe

### TG-C induced IL-10 expressing macrophages in MIA-induced OA rats

**Fig. 3:**
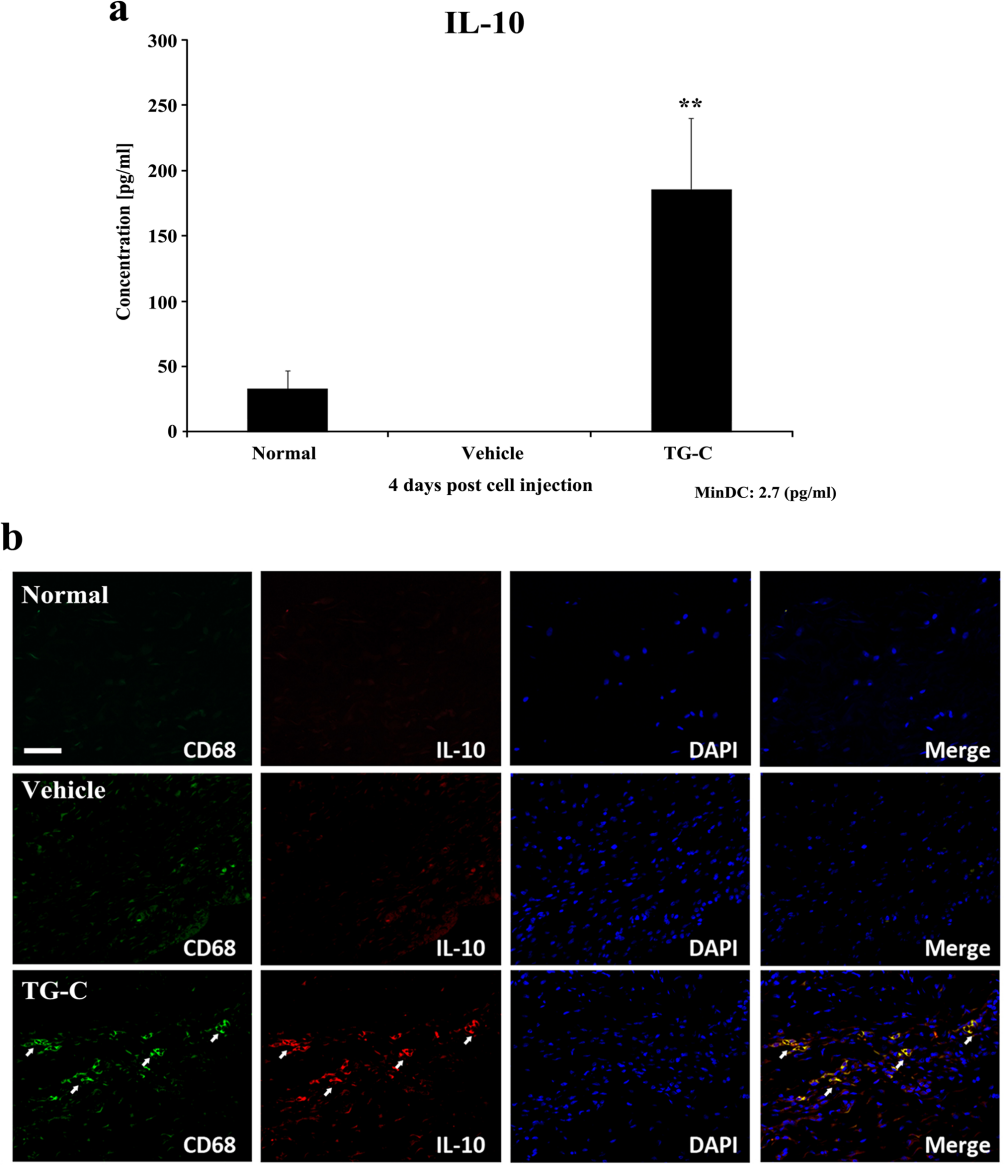
Treatment with TG-C induces the expression of IL-10 in the OA knee joints. Synovial lavage fluid or membranes were harvested at day 4 post-treatment for either a cytokine assay or immunofluorescence. **a** The expression of IL-10 was measured in each treatment group (*n* = 6 per group). The difference was considered statistically significant if *P* < 0.05 (*) or *P* < 0.001 (**), from the vehicle. **b** Synovial membranes were double immunofluorescent stained with anti-CD68 as a macrophage marker or anti-IL-10 antibodies. The white arrow indicates co-localization of CD68 with IL-10 and is shown only in the TG-C treatment group. The scale bar indicates 50 μm

OA is predominantly considered to be a disease caused by mechanical damage (Felson 2013), but recent studies have determined that OA is also as an inflammatory disease (Berenbaum 2013; van den Bosch 2019) as the mechanically induced microtrauma drives low-grade inflammation (Scanzello 2017). Since MIA induces an inflammatory micro-environment in the knee joint, it may be inferred that the analgesic and regenerative effects of TG-C are based on the regulation of the inflammatory micro-environment. To verify that anti-inflammatory factors were induced by TG-C, we analyzed the expression of various cytokines using a Luminex multiplex assay in synovial lavage fluid. We noted that the expression of IL-10 was increased significantly on day 4 after TG-C treatment (Fig. 3a). IL-10 has been reported to have anti-inflammatory activity by modulating immune cells (Dengler et al. 2014; Plunkett et al. 2001; Soderquist et al. 2010; Zheng et al. 2014). Based on these results, we postulated that TG-C can alter the immunological micro-environment in the knee joint of the MIA-induced OA rat model. Among the various types of immune cells, macrophages are known to contribute to the development of OA pathology (Bondeson 2010; Bondeson et al. 2006, 2010, 2001; Goldring 1999; Pelletier et al. 1991). IF staining for CD68, a cell-surface marker of macrophages, showed that recruitment of macrophages in the synovial membrane was elevated in both the vehicle and TG-C groups (Fig. 3b). In contrast, IL-10 expression was highly up-regulated in the TG-C group compared to the normal and vehicle groups. Co-expression of IL-10 with CD68 was only increased in the TG-C group (Fig. 3b, bottom panel). These results suggest that TG-C may affect the abundance, activity and immunoregulatory function of macrophages.

### TG-C induces polarization of M2 macrophage

**Fig. 4:**
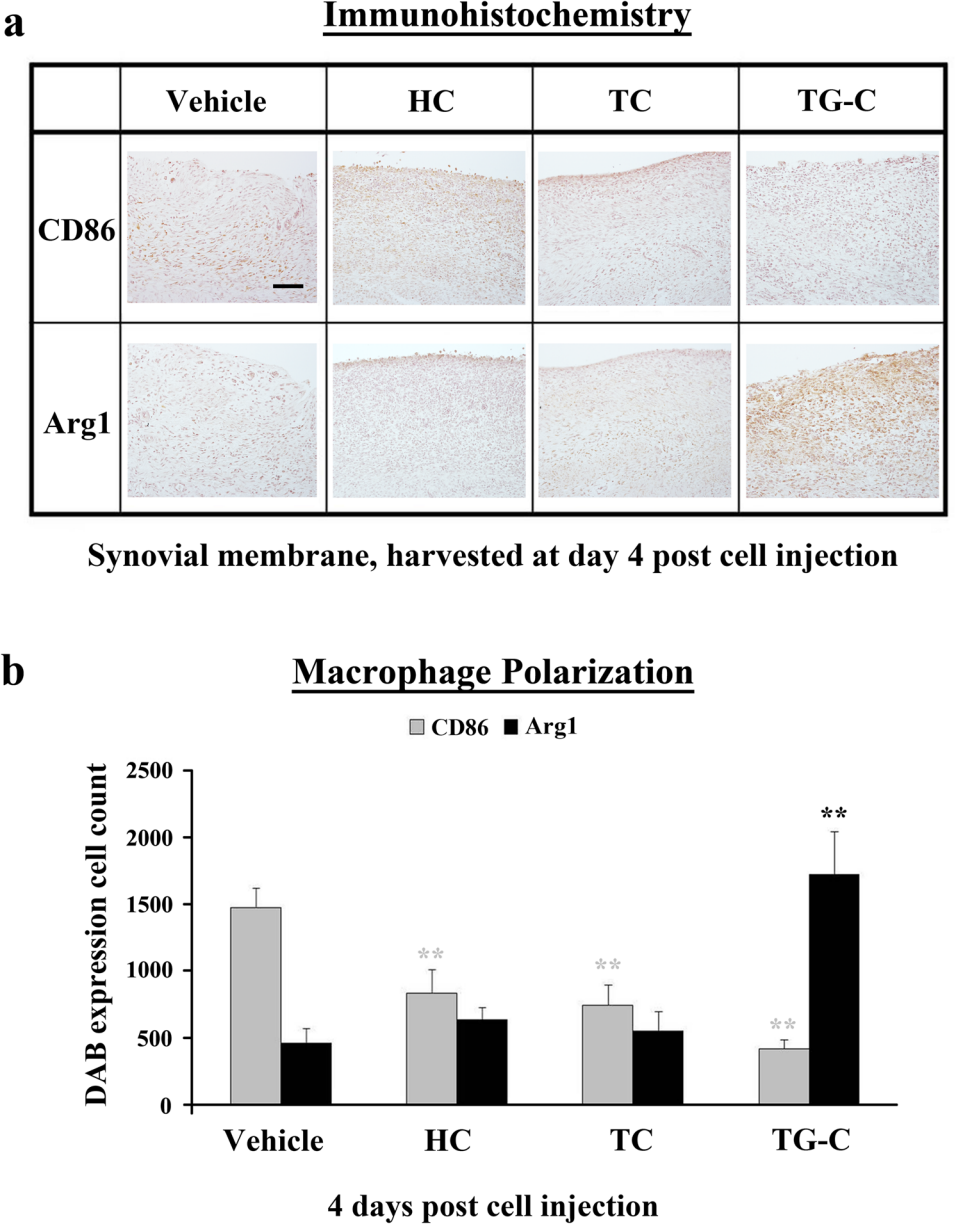
Arginase 1, M2 macrophage specific marker is predominantly expressed following TG-C treatment. **a** At day 4 post-treatment, synovial membranes were stained with anti-CD86 (M1 macrophage marker) or anti-arginase 1 (M2 macrophage marker) antibodies. **b** Distribution of CD86 and Arg1-positive cells following TG-C or individual component cell treatment. CD86 was highly expressed in the vehicle group and decreased in the other treatment groups. Arginase 1 was highly expressed only in the TG-C-treated group, despite the rats having received an injection of MIA. Values represent the mean ± SEM of experimental triplicates. The difference was considered statistically significant if *P* < 0.05 (*) or *P* < 0.001 (**), from the vehicle. All the histology data are representative of three independent experiments, *n* = 24, 6 rats per group. The scale bar indicates 100 μm

To determine whether TG-C can modulate macrophage polarization, we investigated the expression of CD86 as an M1 macrophage specific marker and arginase 1 (Arg 1) as an M2 macrophage specific marker (Abumaree et al. 2013; Medbury et al. 2013). In the vehicle group, expression of CD86 was significantly higher than the other groups (Fig. 3a, upper panel). In contrast, the expression of CD86 was reduced in all the TG-C-treated rats, and the expression of Arg1 was significantly increased only in the TG-C group (Fig. 4a, lower panel). We quantified the polarization of the macrophages among the groups and the data are summarized in Fig. 4b (CD86; vehicle vs TG-C; *P* < 0.001, Arg1; vehicle vs TG-C; *P* < 0.001).

**Fig. 5:**
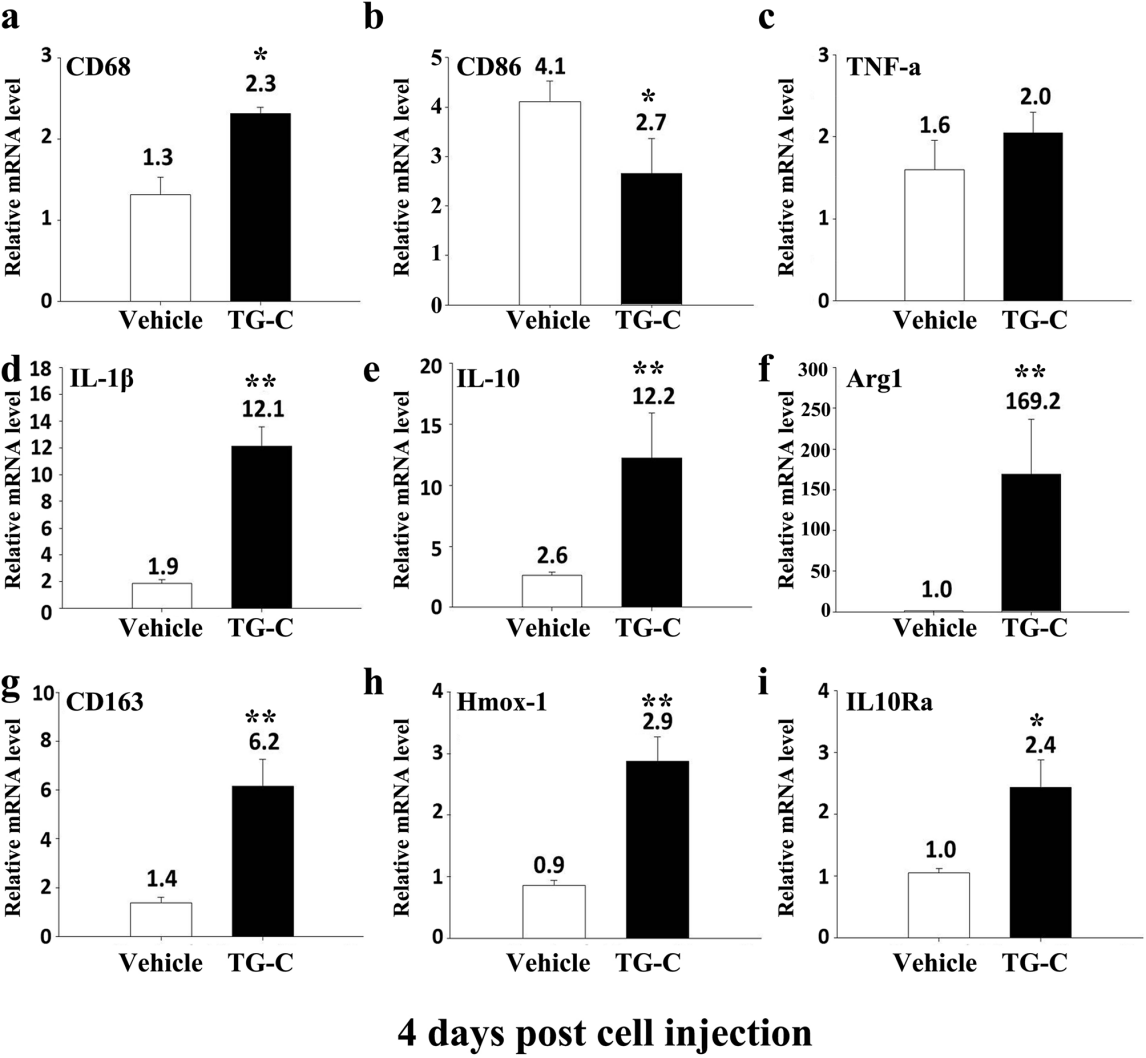
M1 and M2-specific gene expression profiles in rat MIA-induced OA knee joints. Knee joints were collected at day 4 post-treatment and RNA from the synovial membrane was prepared. Subsequent quantitative RT-PCR for the macrophage phenotypes or inflammatory cytokine-related genes was performed. The indicated M1- or M2-specific marker genes and cytokine gene expression profiles were analyzed (*n* = 12, 6 rats per group). The difference was considered statistically significant if *P* < 0.05 (*) or *P* < 0.001 (**), from the vehicle

To further characterize the macrophage phenotypes, gene expression profiles of M1 and M2 macrophage markers were examined by quantitative RT-PCR (qPCR) (Fig. 5). Expression of CD68 mRNA was increased after TG-C treatment (Fig. 5a), consistent with the IHC data shown in Fig. 2. The level of CD86 was decreased (Fig. 5b) and the TNF-α level was unchanged in the TG-C group (Fig. 5c). Expression of the pro-inflammatory cytokine IL-1β mRNA was also increased in the TG-C group (Fig. 5d); however, there was no statistically significant difference in protein levels. In contrast, expression of the anti-inflammatory cytokine IL-10 mRNA was significantly increased following TG-C treatment (Fig. 5e). qPCR was used to confirm that the M2 macrophage markers Arg 1, CD163, interleukin 10 receptor alpha subunit (IL-10RA), and heme oxygenase 1 (Hmox-1) (Weis et al. 2009) were highly up-regulated in the TG-C group (Fig. 5f–i). These results demonstrate that TG-C treatment induced an M2 macrophage-dominant micro-environment in the MIA-induced OA knee joint.

### IL-10 and TGF-β1 play a key role in the efficacy of TG-C

**Fig. 6:**
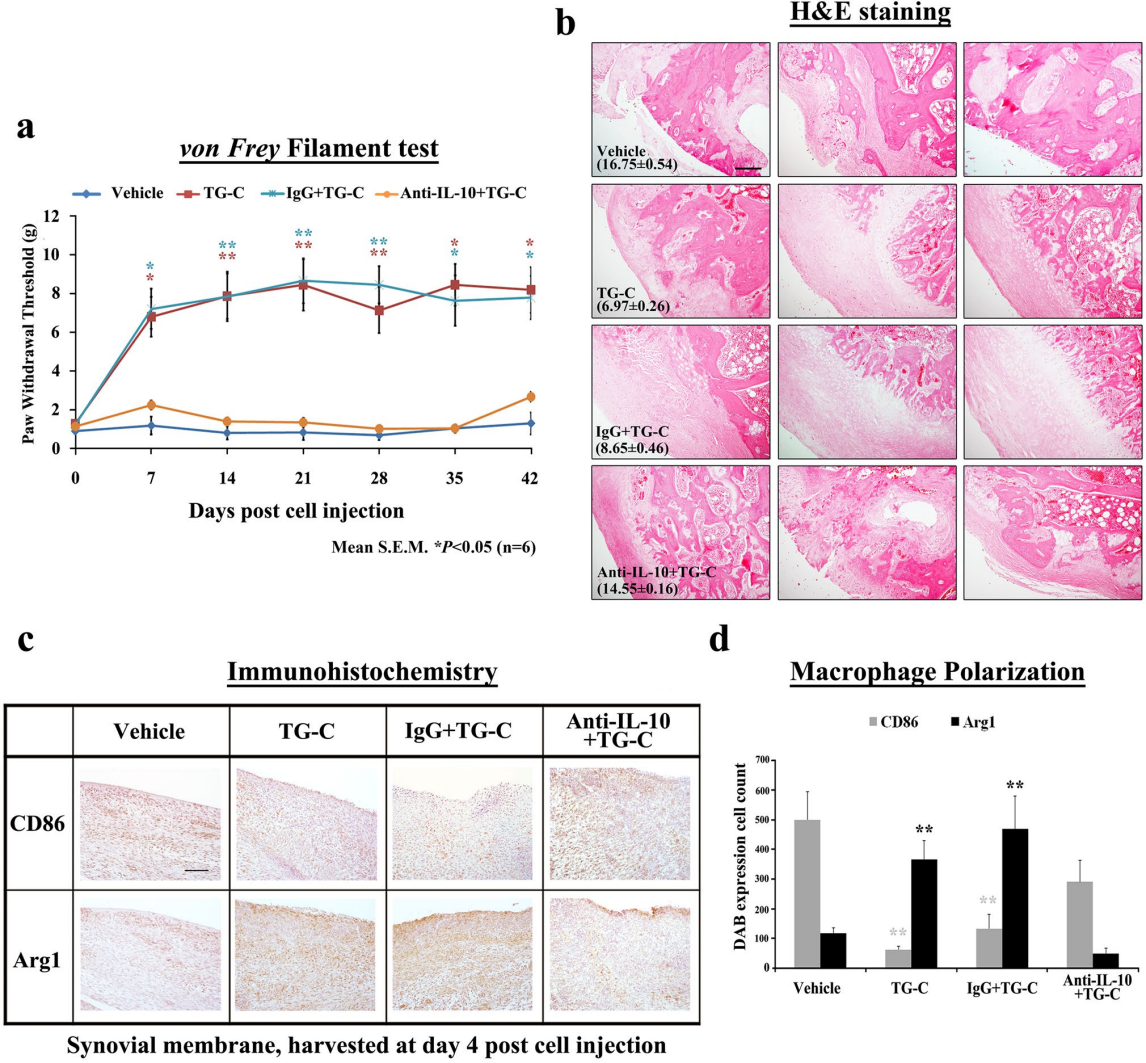
Neutralization of IL-10 attenuates the pain relief and cartilage improvement effects of TG-C. Either the neutralizing anti-IL-10 antibody (anti-IL-10) or isotype control antibody (IgG) group was injected into the knee joints on the same day as TG-C or vehicle treatment. Antibodies were administered once more at day 3 after TG-C or vehicle treatment. **a** The TG-C or TG-C + IgG-treated (IgG) groups induce analgesic effect as measured by the *von Frey* filament test. The analgesic effect of TG-C was eliminated by neutralization of IL-10. Representative results from three independent *von Frey* filament tests are shown (*n* = 24, 6 rats per group). The difference was considered statistically significant if *P* < 0.05 (*) or *P* < 0.001 (**), from the vehicle. **b** H&E staining and grades according to the Mankin’s scoring system also showed that there was no change in terms of structural improvement in the cartilage of the TG-C + anti-IL-10-treated group compared with the vehicle group, which was harvested at day 42 after TG-C treatment. **c** Induction of M1/M2 macrophage polarization was evaluated by immunostaining for CD86 and arginase 1 in synovial membranes harvested at day 4 post-TG-C treatment (*n* = 24, 6 rats per group). **d** Quantitative analyses of immunostaining indicated that there was no significant difference between the vehicle group and the TG-C + anti-IL-10-treated groups. Values represent the mean ± SEM of experimental triplicates. The difference was considered statistically significant if *P* < 0.05 (*) or *P* < 0.001 (**), from the vehicle. The scale bar indicates 100 μm

IL-10 and TGF-β1, which are induced following TG-C treatment, are known to be involved in M2 polarization (Cao et al. 2010). To examine whether these cytokines are critical for the efficacy of TG-C, neutralizing antibodies against each cytokine were injected into the knee joints. The MIA-induced OA model treated with IL-10-neutralizing antibodies (Fig. 6a, orange) showed a pain response similar to that seen in the vehicle group, which was maintained until the end of the observation period (Fig. 6a). In contrast to the IL-10 neutralizing antibody, the isotype control antibody (IgG) had no effect on the pain relief of TG-C (Fig. 6a, sky blue, vehicle vs TG-C + IgG; *P* < 0.001). To investigate the role of IL-10 on the structural improvement effect of TG-C, the knee joints were isolated from the rats and histological analysis was performed. The improvement of cartilage structure in the TG-C group was blocked by the neutralizing antibodies against IL-10 (Fig. 6b). Moreover, immunostaining showed that neutralization of IL-10 abrogated the Arg1 induction activity of TG-C (Fig. 6c). In contrast, IgG had no effect on TG-C induced macrophage polarization. These results are summarized quantitatively in Fig. 6d.

**Fig. 7:**
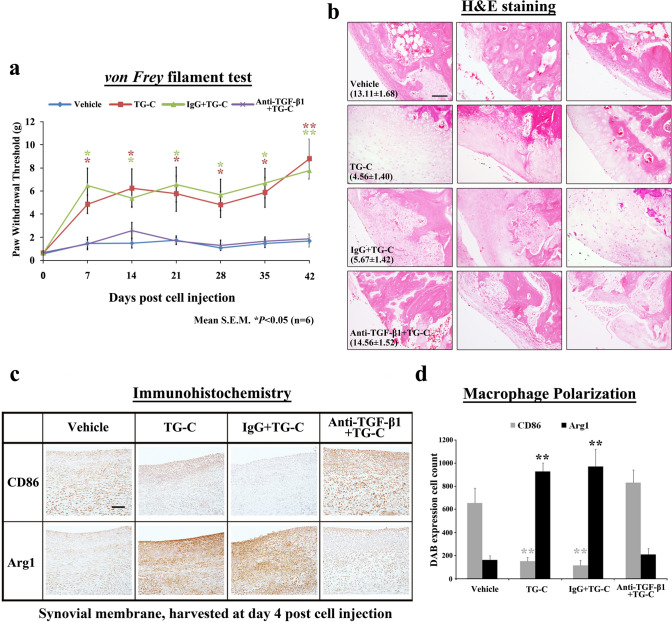
Neutralization of TGF-β1 attenuates pain relief and cartilage improvement effects of TG-C. **a** The *von Frey* filament test showed that the TG-C or TG-C + Isotype IgG-treated (IgG) groups exhibited a pain relief effect. Analgesic effect of TG-C disappeared following neutralization of TGF-β1. Representative results from three independent *von Frey* filament tests are shown (*n* = 24, 6 rats per group). The difference was considered statistically significant if *P* < 0.05 (*) or *P* < 0.001 (**), from the vehicle. **b** In addition, H&E staining and grades according to the Mankin’s scoring system also showed that there was no change in the structural improvement of the cartilage of the TG-C + anti-TGF-β1-treated group compared with the vehicle group, which was harvested at day 42 post-TG-C treatment. **c** Induction of M1/M2 macrophage polarization was evaluated by immunostaining for CD86 and arginase 1 in synovial membrane harvested at day 4 post-TG-C treatment (*n* = 24, 6 rats per group). **d** Quantitative analyses of immunostaining indicated that there was no significant difference between the vehicle group and the TG-C + anti-TGF-β1-treated group. Values represent the mean ± SEM of experimental triplicates. The difference was considered statistically significant if *P* < 0.05 (*) or *P* < 0.001 (**), from the vehicle. The scale bar indicates 100 μm

Neutralization of TGF-β1 also showed similar effects; the anti-TGF-β1-treated group showed similar pain responses to those seen in the vehicle group, whereas the IgG group had no effect on the pain-relieving effects of TG-C (Fig. 7a). Also, structural improvement induced by TG-C was blocked by the neutralizing antibodies against TGF-β1 (Fig. 7b). Neutralization of TGF-β1 blocked the upregulation of Arg1 and induced downregulation of CD86 in the TG-C group (Fig. 7c, d). In summary, these results suggest that IL-10 and TGF-β1 play key roles in the function of TG-C by inducing M2 macrophage polarization.

## Discussion

In this study, we examined the mechanism underlying the action of TG-C to determine whether it is a novel DMOAD that targets the inflammatory micro-environment in the OA synovial joint. We found that TG-C provided pain relief from day 7 post-treatment until the end of the observation period (Fig. 2a) as demonstrated using the *von Frey* filament test. Moreover, TG-C generated hyaline cartilage, which is predominantly composed of type II collagen and is the main constituent of normal joints, rather than type I collagen which is found in other connective tissues and fibrocartilage (Fig. 2b–d). This is important for improved biomechanical joint function and serves as a clinical endpoint.

After evaluating the long-term efficacy, we observed that the expression of IL-10 in the synovium was increased 4 days after treatment of TG-C. Since OA pathogenesis involves the upregulation of inflammatory responses, it is possible that IL-10 is the source by which TG-C combats the MIA-induced OA micro-environment. Interestingly, IL-10 correlated with the increased CD68, a marker of macrophages, in the TG-C group (Fig. 3). Macrophages can be categorized into two distinct subsets (M1 and M2) depending on their activation phenotypes (Ferrante and Leibovich 2012; Jiang et al. 2014). M1 macrophages express high levels of TNF-α, IL-12, IL-23, and low levels of IL-10; this leads to Th1 and Th17 immune responses, which can cause tissue damage (Dai et al. 2015). In contrast, M2 macrophages express high levels of IL-10 and low levels of IL-12 and promote tissue remodeling by reducing inflammation (He and Marneros 2013; Mankin 1982; Zhang et al. 2016). Combinations of IL-4, IL-10 and TGF-β have been shown to induce M2 macrophages (Cao et al. 2010; Mia et al. 2014). In this study, M1 and M2 macrophages were both present in the MIA-induced OA model; however, the level of M1 macrophages was higher (Fig. 4b). In contrast, treatment with TG-C increased the expression of M2 macrophage specific markers, including CD163, Arg1, IL-10RA and Hmox-1 (Fig. 5). This is important, as the M2 dominant micro-environment may play an important role in the improvement of OA symptoms. The imbalance of the M1/M2 macrophage ratio is associated with disease progression in various inflammatory diseases (Funes et al. 2018; Hristodorov et al. 2015; Parisi et al. 2018). In knee OA patients, the imbalanced ratio of M1/M2 macrophages was observed and the degree of this imbalance was associated with the severity of the knee OA. Therefore, the re-balancing of the M1/M2 macrophage ratio has been suggested as a novel therapeutic approach, particularly in the inflammatory and metabolic phenotypes of knee OA (Liu et al. 2018; Mobasheri et al. 2017; Van Spil et al. 2019). In this regard, the change toward the M2-dominant micro-environment by TG-C treatment reflects a reduction in inflammation and indicates the possibility of joint recovery, structure restoration and genuine disease modification. For instance, arginine metabolism is critical for the regulation of mammalian immune responses (Munder 2009) and expression of Arg1 and the resulting depletion of l-arginine is a strong immunosuppressive pathway of the immune system (Bronte and Zanovello 2005). Several studies have demonstrated that l-arginine deficiency leads to an impaired T-cell immune response. Boutard et al. demonstrated that TGF-β upregulates arginase activity in rat peritoneal macrophages and Durante et al. has also demonstrated that TGF-β stimulates l-arginine metabolism by inducing both Arg1 mRNA and arginase activity (Boutard et al. 1995; Durante et al. 2001). These studies support our findings as the changes induced by TG-C promotes an anti-inflammatory micro-environment in the OA knee joint.

Our analysis of macrophage phenotypic markers suggests polarization of macrophages from M1-dominant to M2-dominant following the TG-C injection (Figs. 4, 5). Importantly, neutralization studies indicate that both IL-10 (Fig. 6) and TGF-β1 (Fig. 7) are required for this effect, as well as for the reduction in pain and histological features of OA. This is consistent with our observations as neither HC nor TC alone showed independent OA treatment potential in the MIA model (Fig. 2a). Moreover, the neutralization studies strongly suggest that the induction of an M2 macrophage-dominant synovial micro-environment is key to the efficacy of TG-C in the arthritic knee joint.

The limitations of this study are as follows. Firstly, although we broadly classify macrophages into M1 and M2 types, we do recognize that M2 macrophages can be further divided into several phenotypic subtypes and the function of each subtype is slightly different. Subsequent studies on the population of M2 macrophages induced by TG-C and the long-term stability of their anti-inflammatory phenotype will be needed. In addition, even though CD86 is predominantly expressed in M1 macrophages, it is also expressed in dendritic cells and lymphocytes. Therefore, to accurately investigate the immune-regulating micro-environment induced by TG-C, further cellular characterization using flow cytometry and quantification of cell surface markers is necessary. Secondly, we found that the effect of TG-C was linked to M2 macrophages, but it is not certain that the therapeutic effect is directly induced by M2 macrophages or whether M2 macrophages function to restore homeostasis within an inflamed joint, which subsequently creates a more favorable micro-environment for the release and action of another factor or a combination of other factors that may synergistically mediate anti-inflammatory effects and symptom relief. Despite these limitations, our results support TG-C as a DMOAD candidate, as it provides pain relief and structural improvement in OA joints, which are key requirements of a novel DMOAD.

In summary, the present study indicates that TG-C can significantly reduce both pain and cartilage degeneration in an MIA-induced OA rat model by promoting M2 macrophage polarization through IL-10 and TGF-β1. After application of TG-C to the knee joint cavity, M2 macrophages promoted an anti-inflammatory micro-environment in the knee joints and contributed to structural improvement and analgesic effects. Although further studies are needed to clarify the prevailing mechanisms and identify any other synergistic factors, our study together with the previous clinical trial results demonstrate that TG-C is a DMOAD candidate.

## Abbreviations

DAB: 3,3′-Diaminobenzidine
DAPI: Diamidino-2-phenylindole hydrochloride
DMOAD: Disease-modifying osteoarthritis drug
DNA: Deoxyribonucleic acid
HC: Human chondrocyte
MIA: Monosodium-iodoacetate
MRI: Magnetic resonance imaging
OA: Osteoarthritis
PBS: Phosphate-buffered saline
RNA: Ribonucleic acid
RT-PCR: Real-time polymerase chain reaction
TC: GP2-293 protein packaging cells engineered to overexpress TGF-β1
TLR: Toll-like receptor
TG-C: TissueGene-C
TGF-β1: Transforming growth factor β1
